# Adoptive cell therapy for cancer: combination strategies and biomarkers

**DOI:** 10.3389/fimmu.2025.1603792

**Published:** 2025-08-01

**Authors:** Shiyu Liu, Weibo Jiang, Jiyao Sheng, Lixuan Wang, Mengying Cui

**Affiliations:** ^1^ Department of Hepatobiliary and Pancreatic Surgery, The Second Hospital of Jilin University, Changchun, Jilin, China; ^2^ Orthopedic Medical Center, The Second Hospital of Jilin University, Changchun, Jilin, China

**Keywords:** adoptive cell therapy, CAR-T cells, TILs, TCR-T cells, combination therapy, immunotherapy, biomarkers

## Abstract

Adoptive cell therapy (ACT) is a therapeutic approach that involves the isolation, modification, and expansion of immune cells *ex vivo*, followed by their reinfusion into the patient to enhance anti-tumor immune responses. Various forms of ACT have demonstrated promising clinical outcomes across multiple types of cancer. For example, chimeric antigen receptor (CAR)-T cell therapy, tumor-infiltrating lymphocyte (TIL) therapy, and T-cell receptor-engineered T cell (TCR-T) therapy have received approval from the US Food and Drug Administration. However, the clinical application of ACT remains constrained by limited efficacy and potentially life-threatening toxicities. Diminished efficacy may result from an immunosuppressive tumor microenvironment, poor trafficking and infiltration, exhaustion of infused cells, tumor heterogeneity, and antigen escape. To address these challenges, combination strategies have been developed with the goals of enhancing efficacy and managing adverse effects. Conventional treatments and non-ACT forms of immunotherapy have been incorporated into these combination approaches. Biomarkers play an essential role in optimizing ACT strategies and addressing associated complexities. They can aid in candidate selection, assess the quality of ACT products, monitor long-term therapeutic efficacy, manage toxicity, and guide combination regimens. This review briefly outlines six ACT modalities and their common limitations, summarizes current combination strategies, explores potential future regimens, and offers an overview of biomarkers relevant to ACT. These insights provide valuable guidance for the development and clinical implementation of more effective ACT-based therapies, ultimately aiming to improve patient outcomes.

## 1 Introduction

Adoptive cell therapy (ACT) was initially defined as the transfer of lymphocytes to mediate an effector function. In contrast to immunotherapies that enhance the anti-tumor activity of endogenous T cells, ACT uses lymphocytes or other immune cells that are cultured and selected *ex vivo*, thereby avoiding suppressive factors present *in vivo*. Furthermore, *ex vivo* preparation enables the creation of an optimal host microenvironment for the survival of adoptively transferred cells. For example, lymphodepletion with chemotherapy or radiotherapy (RT) reduces competition from endogenous lymphocytes and removes inhibitory factors. Therefore, patients can receive ACT administration in an optimal state (1). Three types of ACT have received approval from the US Food and Drug Administration (FDA): chimeric antigen receptor (CAR)-T cell therapy, T-cell receptor (TCR)-T cell therapy, and tumor-infiltrating lymphocyte (TIL) therapy. However, even the most extensively studied approach—CAR-T cell therapy—faces considerable limitations, particularly in treating solid tumors (2). Ongoing investigations include the development of CAR-based therapies that use immune cells other than T cells, such as natural killer (NK) cells, macrophages (Mφs), and NK T cells. Although these approaches demonstrate potential, challenges remain.

Among the existing challenges, impaired efficacy represents a major concern. Contributing factors include the immunosuppressive tumor microenvironment (TME), poor trafficking and infiltration, exhaustion of infused cells, tumor heterogeneity, and antigen escape. Moreover, ACT may cause severe adverse effects, including cytokine release syndrome (CRS) and immune effector cell-associated neurotoxicity syndrome (ICANS) (3–5). While the mechanisms underlying the weakened response of various T-cell-based therapies may exhibit considerable overlap, the mechanisms of non-T-cell-based ACT require more specific investigation. For instance, NK-like cells can be suppressed by tumor major histocompatibility complex (MHC) -I overexpression (6, 7); cytokine-induced killer (CIK) cell products often demonstrate limited and unpredictable efficacy due to the variable proportions of distinct subsets within the heterogeneous CIK cell population (8–11). Additionally, the manufacturing of Mφ-based products is frequently constrained by severely limited proliferative capacity (12).

**Figure 1:**
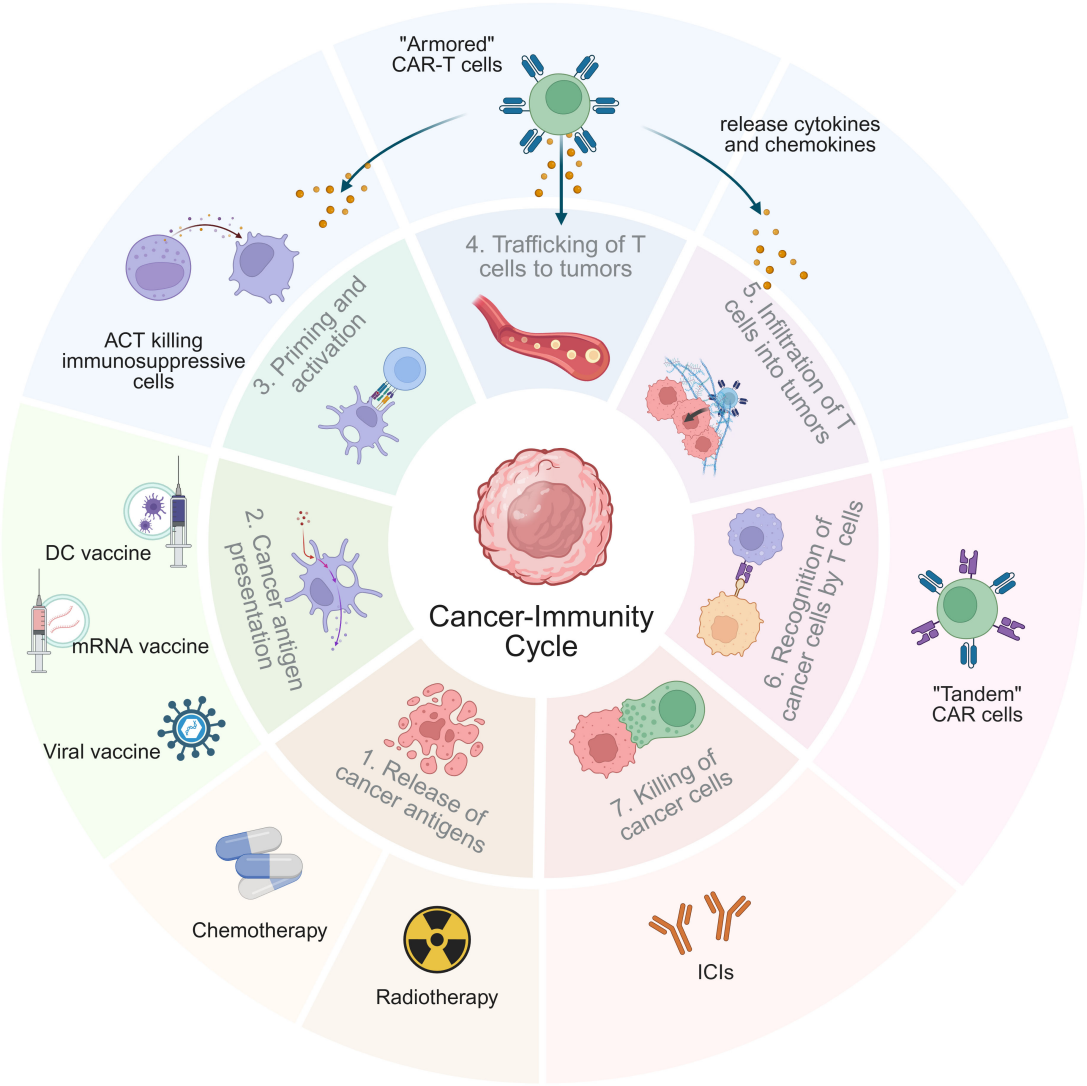
Mechanisms of ACT combination therapy in the tumor-immunity cycle. The tumor-immunity cycle consists of seven key steps, starting from “releasing cancer antigens” (step 1) and ending with “killing cancer cells” (step 7). The mechanisms of ACT-based combination therapies are reflected in one or more steps of the tumor-immunity cycle. Notably, “armored” CAR-T therapy has a connection with steps 3, 4, and 5, highlighting its complex functional mechanism.

Combination strategies enhance ACT through various mechanisms, which can be illustrated in the “cancer-immunity cycle” (**Figure 1**) (13). These include blocking immunosuppressive signaling pathways with immune checkpoint inhibitors (ICIs) to enhance T-cell activity and reduce lymphocyte exhaustion, as well as modulating cytokine networks by upregulating interleukin (IL)-2 and interferon-γ (IFN-γ), while downregulating IL-10, for example (14–16). Chemotherapy and RT can induce direct tumor cell death and the release of damage-associated molecular patterns (DAMPs), thereby augmenting the function of ACT-derived immune cells (17, 18). Rather than directly inducing cytotoxicity, tumor vaccines enhance the presentation of cancer antigens, thereby increasing the reactivity and longevity of adoptively transferred cells (19). Various ACT modalities can act synergistically by leveraging complementary mechanisms to enhance therapeutic efficacy. Additionally, combination therapy can help mitigate CRS and ICANS by modulating immune activation.

Despite these findings, the identification of reliable biomarkers remains necessary to guide clinical oncology decisions, including candidate selection, assessment of ACT product quality, monitoring of therapeutic efficacy, and management of treatment-related toxicities before and after ACT administration.

This review systematically evaluates major ACT modalities—CAR-T, TCR-T, TILs, NK cells, CIK cells, and Mφs—and examines existing combination strategies. The analysis focuses on how these strategies mechanistically address challenges such as TME-mediated immunosuppression, impaired trafficking, limited cell persistence, antigen escape, tumor heterogeneity, and safety concerns. Preclinical and clinical evidence is synthesized for combination approaches involving ACT with ICIs, chemotherapy, RT, tumor vaccines, and cytokines, highlighting how each strategy targets specific limitations. Additionally, this review examines the crucial role of biomarkers—from baseline patient immunological features to ACT product characteristics—in optimizing patient selection, predicting therapeutic responses, and managing toxicities. By integrating mechanistic insights with clinical applications, this review provides a framework for developing personalized ACT-based regimens that maximize efficacy and safety, thereby advancing cancer immunotherapy toward more durable and broadly applicable clinical benefits.

## 2 ACT

ACT represents a potential salvage or alternative strategy of ICI therapy. Notably, hematologic malignancies treated with CAR-T cell therapy have demonstrated objective response rates approaching 100%, an outcome rarely observed with ICIs (20–24). TIL and TCR-T therapies have also produced encouraging results in advanced melanoma and other solid tumors (25–31). Cancers of the gastrointestinal tract, particularly colorectal cancer (CRC), are largely resistant to ICIs; in such cases, ICI therapy is approved only for tumors with microsatellite instability or deficient mismatch repair (32). In contrast, in a phase II study, TCR-T therapy showed a 43.9% (3/7) objective response rate (ORR) in patients with metastatic CRC who were mismatch repair-proficient (28).

Compared with chemotherapy and RT, ACT offers advantages, especially in terms of safety. Chemotherapy and RT rapidly eliminate tumors but often result in systemic toxicities, such as alopecia, severe bone marrow suppression, and gastrointestinal disturbances (33, 34). The core benefit of ACT lies in the selective recognition of tumor cells, which minimizes damage to normal tissues. In addition, some of its severest complications, such as CRS and ICANS, are immunologically manageable (12, 35). TIL and CAR-NK therapies, can reduce treatment burden via personalized preparations or standardized “off-the-shelf” products (7, 25, 36, 37).

### 2.1 CAR-T cells

In 1989, Gross et al. first engineered T cells expressing CARs, which recognize antigens of interest without MHC restrictions (38). Currently, CARs can recognize a wide range of antigens, including tumor-associated antigens (TAAs) and tumor-specific antigens (TSAs) (39–41). The antigen-binding domain of CAR is typically derived from the single-chain variable fragment (scFv) of a monoclonal antibody. Its affinity optimization is needed to balance T-cell activation and toxicity (42, 43). CAR-T evolution from 1st to 5th generation reflects a shift from basic to complex signaling platforms, which improves T-cell activation, proliferation and persistence (44). Current CARs integrate multiple co-stimulatory domains and signaling elements, including components that enable cytokine secretion. Thus far, there have been seven FDA-approved CAR-T products (45–47).

CAR-T therapy has demonstrated remarkable clinical benefits, especially in hematologic malignancies (3). However, its application in solid tumors remains limited owing to challenges such as antigen escape, insufficient trafficking and infiltration, and the immunosuppressive TME. Strategies to enhance efficacy in solid tumors include optimizing target antigen selection, designing CARs that recognize TSAs, improving CAR architecture, modulating the TME, and advancing cell manufacturing and delivery techniques.

CRS and ICANS are common and life-threatening toxicities of CAR-T cell therapy (48). CRS occurs when the activation of CAR-T cells, which release cytokines such as IFN-γ, tumor necrosis factor-α (TNF-α), IL-6, IL-1, IL-10, and granulocyte-macrophage colony-stimulating factor (GM-CSF), is triggered (49). These cytokines activate bystander monocytes and Mφs, amplifying the inflammatory response. TNF-α activates the nuclear factor-kB signaling pathway, inducing the expression of anti-apoptotic and pro-inflammatory genes (50, 51). IL-6 can activate other immune cells, further activating T cells and establishing a “positive feedback loop” (52). Additionally, IL-6 and other cytokines can induce endothelial activation, leading to systemic inflammatory reactions (53). Once the blood-brain barrier (BBB) is disrupted, peripheral cytokines can enter the central nervous system (CNS). Additionally, cluster of differentiation (CD)19 CAR-T cells may recognize CD19^+^ brain mural cells, thereby directly activating CNS immune cells and inducing cytokine release (54, 55).

### 2.2 TCR-T cells

TCR-T cells express engineered TCRs that initiate intrinsic activation upon recognizing specific peptide-MHC (pMHC) complexes. This activation is primarily mediated through the CD3 complex (56, 57). Engineered TCRs with high affinity for pMHC complexes can activate T cells more efficiently and elicit stronger signaling responses (32, 58, 59). Unlike CARs, TCRs recognize both intracellular and surface antigens; they can also respond to lower antigen densities (4).

TCR-T therapy has shown promising efficacy in acute myeloid leukemia (AML) (60). Morelli et al. conducted a first-in-human trial testing a TCR-T product, which recognizes shared *KRAS* and *TP53* mutations in solid tumors. The trial, which involved patients with solid tumors, showed a favorable overall response (OR) lasting up to 6 months (61) (**Table 1**). In August 2024, the FDA approved Tecelra (afamitresgene autoleucel), the first TCR-T product, for the treatment of synovial sarcoma (72).

**Table 1:** Key clinical and preclinical trials of ACT.

Cell type	ACT	Indication(s)	Pts	Key finding(s):	NCT number	Ref.
CAR-T	BCMA CAR-T	r/r MM	128	ORR: 89.1%; CRR: 55.5%; mPFS: 11.8 mos; mOS: 24.8 mos	NCT04196413	(62)
	CD19 CAR-T (Obe-cel)	B-ALL	127	ORR: 77% (95% CI: 67%-85%); CRR: 55% (95% CI: 45%-66%); mOS: 15.6 mos; 12-mo OS rate: 61.1%	NCT04404660	(45)
	CD19 CAR-T (Axi-cel)	LBCL	40	CRR: 78% (95% CI: 62-90); ORR: 89% (95% CI: 75-97); mPFS: NR; 12-mo PFS rate: 75% (95% CI: 55-87)	NCT03761056	(63)
	TRBC1 CAR-T	r/r PTCL	10	ORR: 66.6%; CRR: 40%	NCT03590574	(64)
TCR-T	TCR-T	NSCLC, Colorectal cancer, Pancreatic cancer	3	Manageable safety profile; PR in 1 pt, SD in 1 pt, PD in 1 pt; TCR-T persistence observed	NCT05194735	(61)
	CD19 γ/δ TCR-T (ET019003)	r/r DBCL	8	ORR: 87.5%; CRR: 75%; 3-yr OS: 75.0% (95% CI: 31.5-93.1); 3-yr PFS: 62.5% (95% CI: 22.9-86.1); 3-yr DOR: 71.4% (95% CI: 25.8-92.0)	NCT04014894	(65)
TIL	TIL	Advanced Melanoma	168 (84 TIL, 84 ipilimumab)	mPFS: 7.2 mos (95% CI: 4.2-13.1) vs. 3.1 mos (95% CI: 3.0-4.3); mOS: 25.8 mos (95% CI: 18.2-NR) vs. 18.9 mos (95% CI: 13.8-32.6)	NCT02278887	(25)
	TIL (Lifileucel/LN-144)	Metastatic Melanoma	192	ORR: 56% in anti-PD-1 naive vs. 24% in anti-PD-1 refractory; mOS: 28.5 mos vs. 11.6 mos	NCT01993719, NCT02621021	(66)
	TIL (Lifileucel/LN-145)	mNSCLC	28	ORR: 21.4%; 1 CR, 5 PR; 2 ongoing responses at cut-off	NCT03645928	(30)
NK	CAR-NK (NK-92/5.28.z)	Glioblastoma	9	mPFS: 7 wks; mOS: 31 wks; 2 pts showed progression with PFS of 37 wks and OS of 98 & 135 wks	NCT03383978	(67)
	NK	AML	9	ORR: 75%; CRR: 50% at day 28; 2 pts remained in CR >3 months; 1 pt in remission >2 years	NCT03068819	(68)
CIK	CIK	CRC	N/A (preclinical model)	CIK + oxaliplatin enhances apoptosis in CRC cells via the mitochondrial pathway	Preclinical trial	(69)
	CD33 CAR-CIK	AML	N/A (preclinical model)	CD33.CAR-CIK effective against chemoresistant AML in xenograft models	Preclinical trial	(70)
Mφ	CAR-M (CT-0508)	HER2-overexpressing solid tumors	18	Safety, tolerability, cell manufacturing feasibility, trafficking, TME activation, and preliminary evidence of efficacy evaluated; no specific PFS/OS/ORR/CRR data provided.	NCT04660929	(71)

Despite these findings, the complete response rate (CRR) of TCR-T therapy remains low; many patients experience only transient responses followed by relapse. The development of TCR-T therapy faces multiple challenges. Apart from the T-cell exhaustion and immunosuppressive TME hindering CAR-T therapy, TCR-T therapy is limited by the challenge of producing T cells that precisely express TCRs to identify heterogeneous tumor cells (4).

### 2.3 TILs

In 1988, Rosenberg and colleagues reported the first clinical case in which TILs induced regression in a patient with metastatic melanoma (73). TILs possess multiple antigen-recognition abilities and strong tumor-homing properties, excelling in addressing tumor heterogeneity and infiltration. The first product, LN144, has received FDA approval for the treatment of melanoma. TILs also demonstrate efficacy in other ICI-resistant solid tumors, including breast cancer and non-small cell lung cancer (mNSCLC) (30, 74). Shared resistance mechanisms in ICI and TIL therapies include abnormal neoantigen presentation and reduced tumor mutational burden (TMB) (66, 75, 76). It is reasonable to speculate that TIL therapy would show limited efficacy in patients with ICI resistance. Recent studies have shown that ex vivo expanded TILs from ICI-resistant patients can still recognize and eliminate fresh tumor cells in advanced melanoma and mNSCLC. This may result from TILs’ ability to bypass ICI-specific resistance mechanisms, the absence of *in vivo* T-cell suppressors during manufacturing, and the numerical advantage of TIL therapy. However, *ex vivo* expanded TILs derived from ICI-resistant patients with advanced melanoma and mNSCLC are shown to retain the ability to recognize and eliminate fresh tumor digests. This may be attributed to the capacity of TILs to circumvent ICI-specific resistance mechanisms, the absence of *in vivo* suppressive factors during manufacturing, and the numerical advantage of TILs (25, 29, 30, 66). For example, responses to TIL therapy were observed in mNSCLC with programmed death-ligand 1 (PD-L1)–negative, TMB^low^, or *STK11*-mutated, which typically indicate resistance to ICIs (30). Despite these benefits, patients previously exposed to programmed cell death protein 1(PD-1) inhibitors have a lower response rate to TIL therapy, likely owing to shared resistance mechanisms. Thus, if applied as a first-line treatment, therapeutic potential of TIL therapy may be optimized (66). Ongoing clinical trials (NCT05727904, NCT03645928) are currently evaluating the efficacy and safety of regimens that include lifileucel combined with pembrolizumab in ICI-naïve patients.

Despite its potential, TIL therapy faces challenges. It requires efficient identification and isolation of tumor antigen-specific lymphocytes (77). Additionally, the production process of TIL therapies must be tightly controlled and standardized to ensure the quality and efficacy of the cellular products. However, there are still deficiencies in the expansion process and the selection of specific T-cell subsets for TIL, as well as a lack of comparability in production processes between studies. The Study by Albrecht et al. has shown that the use of the Zellwerk ZRP bioreactor enables the automated control of key parameters in the culture process (e.g., temperature, pH, and pO₂), ensuring the stability and consistency of culture conditions (78).

### 2.4 NK cells

NK cells play a crucial role in innate tumor surveillance, providing innate anti-tumor activity without prior sensitization. This makes them ideal for engineering into CAR-NK cells. With favorable safety profiles, rapid action, and potential for “off-the-shelf” allogeneic products (via reduced manufacturing time or costs), they possess enhanced clinical feasibility. Clinical trials have shown promising results (**Table 1**). On the other hand, their safety and sourcing flexibility position them as an alternative to CAR-T (79). NK cells can induce tumor apoptosis even at low numbers, thereby reducing the risk of excessive cytokine release. Moreover, their MHC-unrestricted activation avoids graft-versus-host disease, even when transferred into allogeneic hosts (79).

Like other ACTs, NK therapy is also hindered by TME suppressive factors, notably transforming growth factor-beta (TGF-β). TGF-β suppresses NK cell function by activating SMAD signaling, inhibiting the secretion of perforin, granzyme B, and IFN-γ, and downregulating activating receptors (e.g., NKG2D, NKp30) through the SMAD signaling pathway, which is activated by the TGFβRI/II receptor complex (80, 81). Blocking this pathway via TGFβRII intracellular domain deletion preserves NK function, supporting trials in TGF-β-rich tumors, such as glioblastoma (82, 83). Although NK therapy also faces common ACT challenges, CAR-NK shows promise with innate advantages over CAR-T (84–86).

### 2.5 CIK cells

CIK cells comprise CD3^+^CD56^+^ NK-T cells, CD3^+^CD56^-^ T cells, and CD3^-^CD56^+^ NK cells. These cells are generated by culturing peripheral blood-derived lymphocytes with anti-CD3 antibodies, IL-2, and IFN-γ (87, 88). CIK cells can recognize tumor cells through both MHC-restricted and MHC-unrestricted pathways. This can help CIK cells overcome tumor antigen escape (89).

CIK therapy exerts anti-tumor effects through multiple mechanisms. Classically, NK-like cells exert toxicity when their inhibitory receptors (e.g., KIR family members) fail to bind MHC-I molecules, known as the “missing self” mechanism. It happens when tumors downregulate MHC-I molecule expression to escape immune surveillance (90–92). NKG2D receptors on NK-like cells bind with NKG2D ligands (e.g., MICA/B) on tumors to trigger cytotoxicity. However, this process is impaired when soluble MICA produced by tumors neutralizes NKG2D receptors (93, 94). In such cases, NK-like cells activate compensatory pathways through co-expressed DNAM-1 receptors, which recognize CD155 molecules on tumors, forming a “dual-receptor recognition network” and helping to maintain anti-tumor function (95, 96).

The proportions of different CIK cell subsets may lead to inconsistent treatment outcomes. A higher proportion of CD3+CD56+ or CD4- CIK cells may correlate with a better response or diminished cytotoxicity (8, 9). Compared with solid tumors, CIK cells show limited efficacy due to the TME, and they tend to perform better in killing hematological malignancies (97). Enhanced manufacturing techniques and combination treatment modalities may be needed to improve the outcomes of CIK therapy.

### 2.6 Mφs

Composed of the innate immune system, Mφs possess the capability of phagocytosis. Mφs polarize into pro-inflammatory M1 (promoting tumor killing) and immunosuppressive M2 (facilitating tumor progression) phenotypes. To address the immune escape mechanisms in the TME, such as the CD47/SIRPα “don’t eat me” axis (inhibiting phagocytosis) and TGF-β-driven M2 polarization, CAR-M was designed. In addition, unlike the first-generation CAR-M, which was “copied” from CAR-T, there is now a second-generation CAR tailored specifically for the Mφs to enhance its effectiveness (98). For example, Zhang et al. engineered induced pluripotent stem cell (iMACs)-derived Mφs with CD3ζ-TIR-CAR, which incorporates the intracellular Toll/IL-1 receptor (TIR) domain of Toll-like receptor 4. This CAR design features tandem CD3ζ-TIR dual signaling, enabling iMACs to exhibit both target cell phagocytic ability and antigen-dependent M1 polarization while resisting conversion to the M2 phenotype (99).

Clinical trials have shown that Mφ-based therapies are safe, with minimal side effects (e.g., fever, abdominal discomfort) (100) (**Table 1**). The human epidermal growth factor receptor 2 (HER2) CAR-M product CT-0508 has shown efficacy in refractory HER2^+^ solid tumors receiving conventional treatment (71). Similar to NK cells, which have shorter lifespans and lower cytotoxicity compared to T cells, Mφs also hold potential for developing “off-the-shelf” products. However, Mφs are more difficult to use for ACT than T cells because of their weak proliferative capacity both *in vivo* and *ex vivo* (12).

## 3 Challenges

The TME inhibits the function of adoptively transferred cells through multiple mechanisms, including immune checkpoint (ICP) signaling, infiltration of immunosuppressive cells such as regulatory T (Treg) cells and M2 Mφs, pro-tumor cytokines, and metabolic obstacles (101–104). In the TME, there is a cytokine network that maintains a dynamic balance where pro-inflammatory and anti-inflammatory cytokines regulate immune activation and tolerance (105). Imbalanced expression of cytokines (e.g., IL-6, IL-10, and TGF-β) and chemokines (e.g., chemokine [C-X-C motif] ligand [CXCL], CXCL8, CXCL10, and CXCL12) may promote the recruitment of myeloid-derived suppressor cells (MDSCs) while limiting the infiltration of antigen-presenting cells (APCs) (106–108). Treg cells, MDSCs, and tumor-associated Mφs (TAMs) in TME contribute to this imbalance by secreting excessive TGF-β and IL-10 (109–111).

Adoptively transferred cells often fail to enter the TME and exert only limited function because of inefficient trafficking and infiltration. Inefficient trafficking results from abnormal tumor vasculature, a lack of adhesion molecules (e.g., intercellular adhesion molecule-1 [ICAM-1]) and chemokines, and ‘anergic’ vessels that are unresponsive to inflammatory signals (112–114). Additionally, tumor-associated blood vessels often remain “anergic” to inflammatory stimuli and do not upregulate the ligands necessary for T-cell binding, even after exposure to cytokines such as TNF-α (114). Infiltration of immunocytes into solid tumors is obstructed by the TME and physical barriers, including fibroblast-derived collagen and hyaluronan (112, 113, 115).

As a “living” therapy, one of ACT’s advantages lies in the sustained presence of transferred cells *in vivo*. In clinical settings, exhaustion of infused cells is a key contributor to treatment failure. During *ex vivo* expansion, T cells often undergo a progressive loss of stem cell-like properties, including self-renewal capacity and multipotency. This shift is characterized by reduced cytokine production, and increased expression of inhibitory receptors, such as PD-1 and T-cell immunoglobulin and mucin domain 3 (TIM-3) (116, 117). Additionally, PD-L1 levels are likely to be elevated by iron metabolism reprogramming through the ROS/c-Myc pathway, further enabling immune evasion (118). Metabolic checkpoints (e.g., IDO/TDO depletion of tryptophan) and aberrant Wnt/β-catenin signaling also suppress T-cell function and facilitate immune exclusion (102). Conventional culture methods like IL-2 stimulation promote effector differentiation, whereas IL-7 and IL-15 support oxidative phosphorylation and help maintain a naïve phenotype (119–121).

Tumor cells display extensive genetic and epigenetic variation during proliferation, resulting in pronounced tumor heterogeneity (122, 123). Solid tumors exhibit high antigenic heterogeneity and mutational diversity, which hinders the specific identification by T cells (124, 125). Under immunoselected pressure, tumor cells can evade immune recognition through downregulate or lose target antigens, which is defined as antigen escape. This highlights the function of CAR-T cells, as CARs can recognize them only when the surface antigens reach a certain density. This situation is particularly important for single antigen-specific CAR-T (126, 127). For example, glioblastoma patients treated with epidermal growth factor receptor (EGFR) variant III CAR-T cells showed reduced EGFRvIII expression in post-treatment biopsies (128, 129). TCR-T cells rely on an MHC-restricted mechanism for antigen recognition, which is dependent on the antigen processing and presentation mechanisms in tumor cells. Genetic defects, such as MHC gene mutations or heterozygosity loss, disrupt antigen presentation (130, 131). Epigenetic modifications, such as silencing of MHC-I via downregulation of the melanocyte-inducing transcription factor, also contribute to immune evasion (132). Additionally, post-translational mechanisms, such as NBR1-mediated MHC-I downregulation, further inhibit antigen presentation in pancreatic cancer (133–135).

## 4 Combination therapy

**Figure 2:**
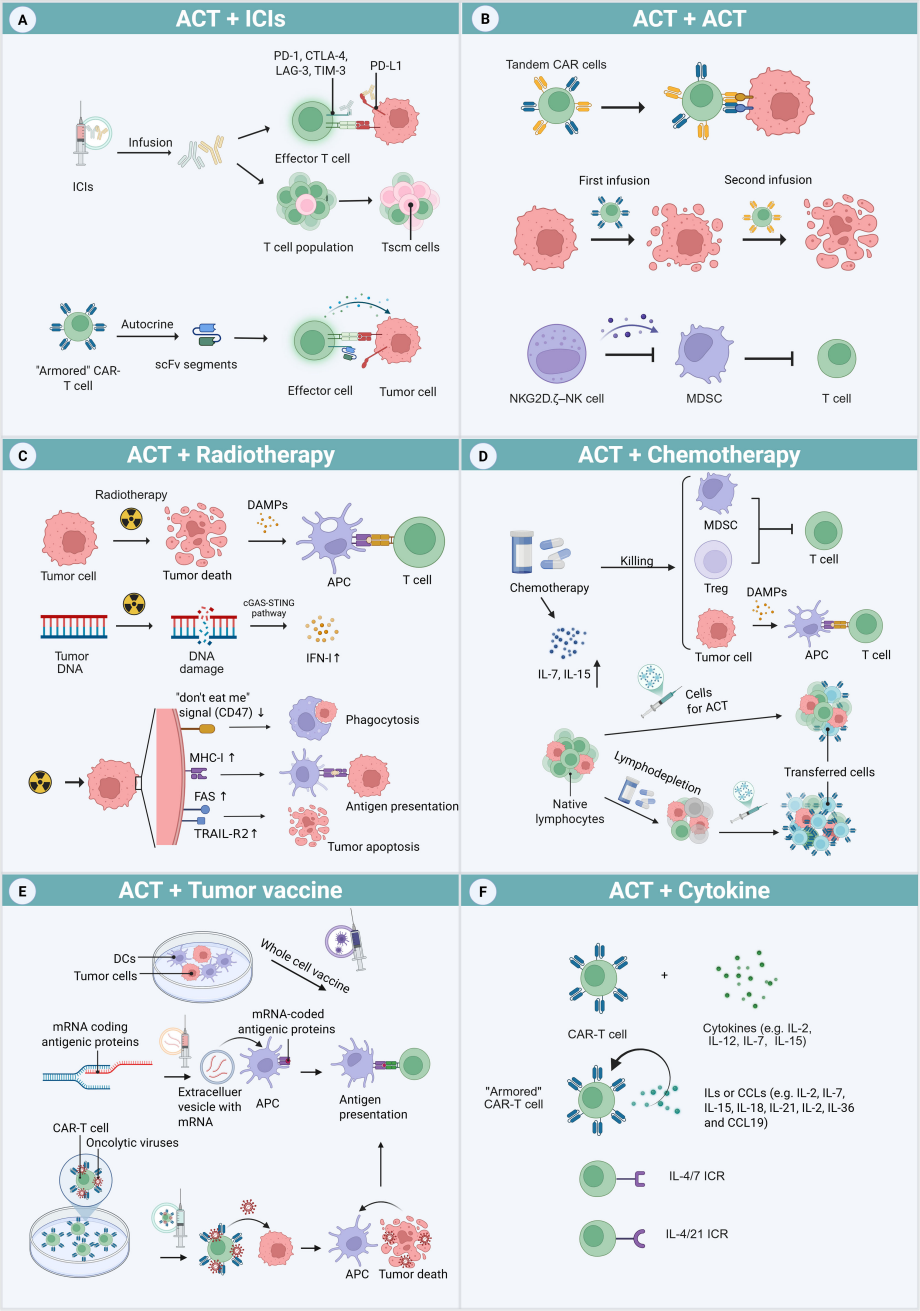
ACT combination therapy approaches. **(A)** ICP signaling pathways can be blocked by exogenous ICIs and endogenous ICI-like scFvs secreted by “armored” CAR-T cells. ICIs can also elevate the proportion of progenitor T cells in T cell clusters. **(B)** Methods to address antigenic heterogeneity include engineering tandem CAR-T cells carrying two types of CARs, and repeated adoptive transfers to further destroy tumor cells. NKG2D.ζ–NK cells function by eliminating MDSCs within the TME. **(C)** Radiotherapy induces tumor cell death and DNA damage, activates the APCs and cGAS-STING pathway, which mediates the production of IFN-I. Additionally, it increases the expression of the “don’t eat me” signals (CD47), MHC-I molecules, and death receptors (FAS and TRAIL-R2) on tumor surfaces. **(D)** Chemotherapy not only kills tumors but also depletes MDSCs and Treg cells, which release DAMPs after cell death. It also increases the levels of IL-7 and IL-15 favorable for transferred cells. Furthermore, chemical agent-based lymphodepletion reduces the survival pressure of transferred cells by eliminating the host’s innate lymphocytes, which compete for growth sources. **(E)** Different tumor vaccines can enhance the presentation of antigen to T cells, including whole-cell vaccines produced by culturing DCs with tumor antigens and mRNA encoding specific antigens. By loading oncolytic virus onto CAR-T cells, tumors release abundant antigens after being killed by the released oncolytic virus. **(F)** The traditional combined strategies of CAR-T cells and cytokines include co-infusion or culture of cells and cytokines. A novel approach involves engineering “armored” CAR-T cells to secrete cytokines or chemokines. Additionally, CAR-T cells can be modified to express inverted cytokine receptors (ICRs), which convert inhibitory signals into activating ones.

The combination of ACTs with other treatments has become a powerful strategy to enhance therapeutic efficacy. Methods that integrate ACT with chemotherapy, RT, or ICIs can substantially augment the overall anti-tumor response. These combination strategies enhance clinical outcomes while supporting a more personalized and comprehensive therapeutic approach (**Figure 2**).

### 4.1 ACT + ICIs

Lymphocyte exhaustion contributes to poor ACT outcomes, and ICIs play a key role in overcoming this barrier. After initial T-cell activation via recognition of tumor antigens, ICPs are upregulated on lymphocytes and tumor cells. ICIs block these inhibitory signaling pathways and restore T-cell activity (136) (**Figure 2A**). In addition, ICIs modulate cytokine expression within the TME. IL-2 and IFN-γ are pivotal cytokines driving CD8^+^ T cell proliferation and activation. PD-1 engagement with PD-L1 markedly inhibits the secretion of these cytokines, a process that ICIs can potentially reverse (14, 15). Tumors often display elevated IL-10 production from Mφs and Treg cells. Notably, PD-1 deficiency in Mφs enhances IL-10 secretion, which in turn suppresses T helper type 1 (Th1) cells. ICIs may disrupt this axis by inhibiting PD-1-mediated IL-10 upregulation, thereby modulating the tumor immunosuppressive milieu (16).

In hematologic malignancies, the combination of PD-1 blockades with CAR-T cell therapy enhances the response rate and CAR-T cell persistence. In a retrospective analysis, patients with relapsed/refractory (r/r) diffuse large B-cell lymphoma (DLBL) and *TP53* mutations received CD19 or CD20 CAR T therapy individually or in combination with PD-1 inhibitors (Sintilimab/Tislelizumab). The results showed that the overall survival (OS) of the CAR-T + PD-1 inhibitors group was not reached, while the OS of the CAR-T group alone was 10.9 months (137). Similar benefits have been observed in cases of leukemia managed by CIK therapy combined with various ICIs (138). In solid tumors, these combination strategies have also yielded encouraging results (139). A phase 2 trial in ICIs-naive advanced melanoma resulted ORR of 63.6% and 5 CRs with TILs + pembrolizumab. After a 17.2-month follow-up, the median response duration remained unreached (29). A Phase 3 study (NCT05727904), still recruiting participants, will assess the efficacy and safety of the TIL product TILVANCE-301 plus pembrolizumab versus pembrolizumab alone for untreated unresectable or metastatic melanoma.

Miller et al. found that among the exhausted CD8^+^ TILs, a small population of progenitor or stem-like T cells (TCF1^+^TIM-3^-^) can differentiate into highly toxic, terminally exhausted TILs. Furthermore, PD-1 blockades may increase their proportion in TILs by promoting proliferation (140). Huang et al. identified a subset of tumor-specific CD8^+^ cells in the tumor-draining lymph nodes, which exhibited typical memory characteristics and anti-tumor effects after adoptive transfer. These cells were identified as responders to PD-1/PD-L1 blockades (141). The outcomes of clinical trials evaluating TIL therapy plus pembrolizumab in patients with various solid tumors have been particularly promising (**Table 2**).

**Table 2:** Key clinical and preclinical trials of ACT combination therapy.

Strategy	ACT	Combination therapy	Indication(s)	Pts	Key finding(s)	Clinical trial number	Ref.
ACT + ICIs	mesothelin CAR-T	pembrolizumab	MPM, metastatic lung cancer and metastatic breast cancer	23	mOS: 23.9 mos; 1-year OS: 83%; 8 patients had SD ≥6 months; 2 had complete metabolic response	NCT02414269	(142)
	CEA CAR-T	(autocrining) PD-1-TREM2 scFv	Colorectal cancer	N/A (preclinical model)	PD-1-TREM2 scFv CAR-T showed enhanced tumor elimination compared to PD-1 scFv CAR-T in mouse model	Preclinical trial	(143)
	TIL	Pembrolizumab	Melanoma, HNSCC, Cervical Cancer	32	ORR: 56.3% (melanoma 87.5%, HNSCC 42.9%, cervical 50.0%); 10/17 responses ongoing at cutoff	NCT03645928, NCT03108495	(139)
	TIL	Pembrolizumab	Melanoma	22	ORR: 63.6% (CR 22.7%, PR 40.9%); mDOR: NR; 10/14 responses ongoing; 8/14 responses ≥12 mos	NCT03645928	(29)
**ACT +ACT**	NKG2D.ζD-NK cell	GD2 CAR-T	Neuroblastoma	N/A (preclinical model)	NKG2D.C-NK cells improved CAR-T cell infiltration and antitumor activity in solid tumors with immunosuppressive TME.	Preclinical trial	(144)
	Tandem CD19/20 CAR-T		r/r B cell NHL	11	mPFS: NR; 12-month PFS rate: 60% (95% CI: 31.27%-83.18%) mOS: NR; 12-month OS rate: 81.82% (95% CI: 52.30%-96.77%)	NCT04723914	(145)
	Tandem CD19/20 CAR-T		r/r B cell NHL	87	MPFS: 27.6 months (95% CI: 11 months to NR) MOS: NR; 12-month OS rate: 79% (95% CI: 69-86)	NCT03097770	(146)
	Tandem CD19/20 CAR-T		r/r B cell NHL	28	mPFS: NR; 12-mo PFS rate: 64% (95% CI: 43-79) mOS: NR; 12-mo OS rate: 71% (95% CI: 51-85)	NCT03097770	(147)
	CD19 CAR-T	CD22 CAR-T	r/r B-ALL	21	ORR: 73% (95% CI: 47%-91%); CRR73% (95% CI: 47%-91%)	NCT02315612	(148)
**ACT + Chemotherapy**	DC/CIK cell	Docetaxel, Pemetrexed	NSCLC	135	mPFS: 5.7 mos; 12-mo PFS: 29.4%; mOS: 17.5; 12-mo OS: 58.2%	NCT03360630	(149)
	CD19 CAR-T	Cyclophosphamide	r/r B-ALL	25	ORR: 75% (95% CI: 53%-90%)	NCT01860937	(150)
**ACT + Radiotherapy**	GPC3 CAR-T	Gamma Knife Radiosurgery	HCC	2	mOS: >8 years; 5-year DFS: 100%	NCT02395250	(151)
	CAR-T (CART19)	LD-TBI	ALL	N/A (preclinical model)	mOS: 41 days (CART19 only); 110 days (LD-TBI + CART19); 28 days (Control)	Preclinical trial	(152)
**ACT + tumor vaccines**	CD19 CAR-T	EPS8- or WT1-targeted DC vaccines	r/r B-ALL	8	mPFS: 489 days; mOS: NR	NCT03291444	(19)
	CMV-CTLs (CAR-GD2)	K562-derived whole-cell vaccine	Neuroblastoma	N/A (preclinical model)	Enhanced antitumor effects in xenograft models; Improved tumor control and survival in vaccinated mice.	Preclinical trial	(153)
	CLDN6 CAR-T	CAR-T-amplifying RNA vaccine	r/r CLDN6+ solid tumors	22	ORR: 33%; CRR: 5%	NCT04503278	(154)
**ACT + cytokines**	CD20 CAR-T	IL-7 and CCL19 (autocrine)	Lung cancer, Pancreatic ductal adenocarcinoma	N/A (preclinical model)	Complete tumor regression in all mice treated with 7x19 CAR-T cells; prolonged survival compared to conventional CAR-T cells.	Preclinical trial	(155)
	Mesothelin CAR-T	IL-2 (autocrine)	Pancreatic cancer, Melanoma)	N/A (preclinical model)	Complete tumor clearance in orthotopic KPC model; increased T-cell infiltration and activation in tumor core.	Preclinical trial	(156)
	PSCA CAR-T	4/7 ICR (co-expression)	Pancreatic cancer	N/A (preclinical model)	Enhanced tumor regression and improved survival in preclinical models; increased T-cell persistence and functionality.	Preclinical trial	(157)
	GPC3 CAR-T	4/21 ICR (co-expression)	HCC)	N/A (preclinical model)	Significant tumor regression and prolonged survival in preclinical models; improved T-cell expansion and anti-tumor activity.	Preclinical trial	(158)

ACT, adoptive cell therapy; Pts, patients; Ref, reference; BCMA, B-cell maturation antigen; CI, confidence interval; DOR, Date of response; LBCL, large B-cell lymphoma; mo, month; mOS, median overall survival; mPFS, median progression-free survival; N/A, not applicable; NR, not reached; PD, progressive disease; SD, stable disease; TRBC1, T-cell receptor beta chain 1; wks, weeks; Yr, year.

Notably, ICI efficacy may be enhanced by ACT. Evidence shows that NKG2D.ζ-D NK cells can eliminate MDSCs, which reduces the effector function of ICIs (144). A novel strategy that modifies cells to secrete ICIs locally represents an alternative approach to exogenous ICIs. Chen et al. developed CAR-T cells capable of secreting a PD-1 scFv, which primarily accumulates at the tumor site alongside the CAR-T cells. This minimizes systemic toxicity associated with widespread ICI distribution (143).

Research concerning CAR-Mφ therapy in combination with ICIs is also ongoing (71). However, further investigations are needed to determine the feasibility of combining ACT with ICIs.

### 4.2 ACT + ACT

Antigen escape remains a significant obstacle to the long-term efficacy and durability of ACT. Under the selective pressure of antigen-specific adoptive cells, tumor cells may downregulate targeted antigens (1). Targeting multiple antigens can help mitigate antigen escape, extend the persistence of ACT, and reduce the likelihood of relapse (**Figure 2B**). One approach involves engineering “tandem CARs,” which incorporate two tumor-specific antigen–targeting scFvs within a single CAR construct. In r/r non-Hodgkin lymphoma (NHL), tandem CD19/CD20 CAR-T cell therapy has produced ORR ranging from 70% to 90%, with a CRR of approximately 70% (145–147). One tri-specific CAR-T product targeting HER2, IL-13 receptor α2 (IL-13Rα2) and EphA2 has shown encouraging results in the treatment of glioblastoma (159). Another strategy involves sequential administration of CAR-T cells targeting different antigens. For instance, reinfusion of CD22 CAR-T cells displayed substantial clinical benefit in patients with r/r B-cell acute lymphoblastic leukemia (B-ALL) who had previously relapsed due to CD19 antigen loss after CD19 CAR-T therapy (148). Additionally, approaches that combine two types of ACT may eliminate both tumor cells and immunosuppressive cell populations. In a preclinical study, CAR-T cells were co-administered with NKG2D.ζ NK cells. The engineered NK cells enhanced the anti-tumor activity of CAR-T cells within solid tumors by depleting MDSCs and secreting pro-inflammatory cytokines (144).

### 4.3 ACT + chemotherapy

Chemotherapy can directly kill tumors, while also depleted Treg cells and MDSCs that inhibit effector T (Teff) cells. It is worth noting that cyclophosphamide and carboplatin have been proven to be more cytotoxic to Treg cells than to Teff cells (160, 161).Furthermore, chemotherapeutic agents induce tumor cell death and promote the release of DAMPs, including adenosine triphosphate, high-mobility group box 1 (HMGB1), and type 1 IFN (IFN-I). These DAMPs contribute to dendritic cell (DC) activation and facilitate antigen presentation to lymphocytes (17) (**Figure 2C**). This form of tumor apoptosis, which involves innate immune responses, is termed immunogenic cell death (ICD). After ICD, DCs and T cells are more likely to accumulate within the TME and secrete TNF-γ (162). A study on ErbB CAR-T cell therapy demonstrated that pretreatment with carboplatin enhanced tumor regression, even at lower CAR-T cell doses (160). Additionally, CIK or DC/CIK therapy plus chemotherapy has been shown to improve OS and progression-free survival (PFS) across various malignancies (89).

Chemical agent-based lymphodepletion before cell infusion is a critical step in ACT, as it enhances the tumor reactivity and persistence of transferred lymphocytes. By depleting endogenous lymphocytes, lymphodepletion reduces competition for cytokines and resources, thus facilitating interactions between infused T cells and tumor antigens (1). In a study by Curran et al., preconditioning lymphodepletion via high-dose cyclophosphamide led to enhanced therapeutic responses and CAR-T cell expansion without greater toxicity when combined with CD19 CAR-T therapy (150).

### 4.4 ACT + RT

RT enhances the efficacy of CAR-T cell therapy through several mechanisms (**Figure 2D**). RT promotes ICD by increasing DAMP production and modulating the TME. After radiation-induced DNA damage, activation of the cyclic guanosine monophosphate/adenosine monophosphate synthase–stimulator of interferon genes (cGAS-STING) pathway leads to upregulation of IFN-I expression. Local RT in combination with ACT has been shown to improve T-cell infiltration by elevating chemokine and cytokine levels (18, 152) (18). Moreover, RT can downregulate the expression of CD47 (the “do not eat me” signal) while simultaneously upregulating MHC-I molecules and death receptors on tumor cells, thus promoting APC activation and enhancing immune recognition.

Indications for RT in the context of CAR-T cell therapy include reducing recurrence or progression at high-risk sites, serving as a palliative intervention, and managing tumor deposits located near vital structures (163). RT can be administered either before or after CAR-T cell infusion. Pre-treatment RT may reduce tumor burden in high-risk cases. For example, two patients with rapidly progressing inferior vena cava tumor thrombus underwent Gamma Knife treatment, followed by infusion of glypican-3 (GPC3) CAR-T cells, which resulted in favorable clinical outcomes (151). Salvage RT is increasingly used in cases of post-CAR-T disease progression. Patients with DLBL (164) or multiple myeloma (MM) (165) who experience local recurrence after CAR-T cell therapy may benefit from subsequent RT.

### 4.5 ACT + tumor vaccines

Tumor vaccines, administered in the form of whole cells, oncolytic viruses or molecular agents such as peptides or RNA, can enhance ACT by priming cancer antigen presentation. This process increases the reactivity and persistence of adoptively transferred cells (**Figure 2E**).

Through presenting TAA to T cells, DC vaccines activate and promote the proliferation of those cells (166). The administration of DC vaccines cultured with EGFR pathway substrate 8 (Eps8)-derived peptide or Wilms’ tumor 1 protein (WT1) after CD19 CAR-T therapy has been shown to increase the proportion of central memory T (Tcm) cells, stimulate CAR-T cell expansion, and improve therapeutic efficacy in r/r B-ALL (19). Similar effects were also observed with the combination of DC vaccines and CIK therapy (89). In addition to DCs, tumor cells themselves can be modified to serve as vaccines. For instance, a whole-cell vaccine based on K562 cells engineered to express cytomegalovirus (CMV)-derived phosphoprotein 65 facilitates (pp65) CAR-T cell proliferation and activation by presenting CMV 65 and co-expressing immunostimulatory molecules (e.g., CD40L and OX40L) (153).

Oncolytic viruses selectively infect and lyse tumor cells, but they can also stimulate systemic immune responses after intratumorally injection. In a study by Evgin et al., mice received CAR-T cells preloaded with oncolytic viruses such as vesicular stomatitis virus or reovirus bound to specific receptors. Upon arrival at the tumor site, the viruses replicated within tumor cells and triggered localized inflammation. This approach increased T-cell specificity and potency, thereby enhancing proliferation and anti-tumor activity (167).

Compared with conventional protein- or peptide-based cancer vaccines, mRNA vaccines offer advantages in both personalization and large-scale production. They address the unique therapeutic needs of individual patients without reliance on specific human leukocyte antigen haplotypes (168). Promising outcomes were observed regarding the combination of oncofetal antigen claudin 6 (CLDN6) CAR-T cell therapy and an amplifying RNA vaccine in solid tumors. The modified nanoparticulate vaccine, CARVac, incorporates CAR target antigen–encoding mRNA encapsulated in lipid-based structures, known as RNA-lipoplexes. Upon administration, the vaccine facilitates systemic delivery of the RNA to APCs in lymphoid tissues (154).

### 4.6 ACT + cytokines

Cytokines serve as signaling molecules that regulate immune cell homeostasis and orchestrate signal-dependent immune responses (169). In the context of conventional ACT, cytokines such as IL-2, IL-7, IL-12, and IL-15 have been used through *ex vivo* preconditioning or *in vivo* co-administration (170) (**Figure 2F**). In CAR-T cell therapy, two innovative strategies use genetic engineering to enhance therapeutic function. The first strategy involves engineering CAR-T cells to produce specific cytokines autonomously. These fourth-generation CAR-T cells, or “armored” CAR-T cells, secrete cytokines (IL-2, IL-7, IL-15, IL-18, IL-21, IL-36) or chemokines (e.g., CC motif chemokine ligand [CCL]19) to improve anti-tumor activity in the TME (170). The second strategy involves modifying the architecture of cytokine receptors to alter intracellular signaling pathways. For example, by fusing the extracellular domain of the IL-4R with the intracellular domain of the IL-7R, researchers developed a novel inverted cytokine receptor (ICR), termed IL-4/7 ICR. Co-expression of this ICR with a CAR in T cells leads to enhanced expansion and anti-tumor activity (157). Similar results were observed with IL-4/21 ICR, which combines the IL-4R and IL-21R (158).

## 5 Biomarkers

**Figure 3:**
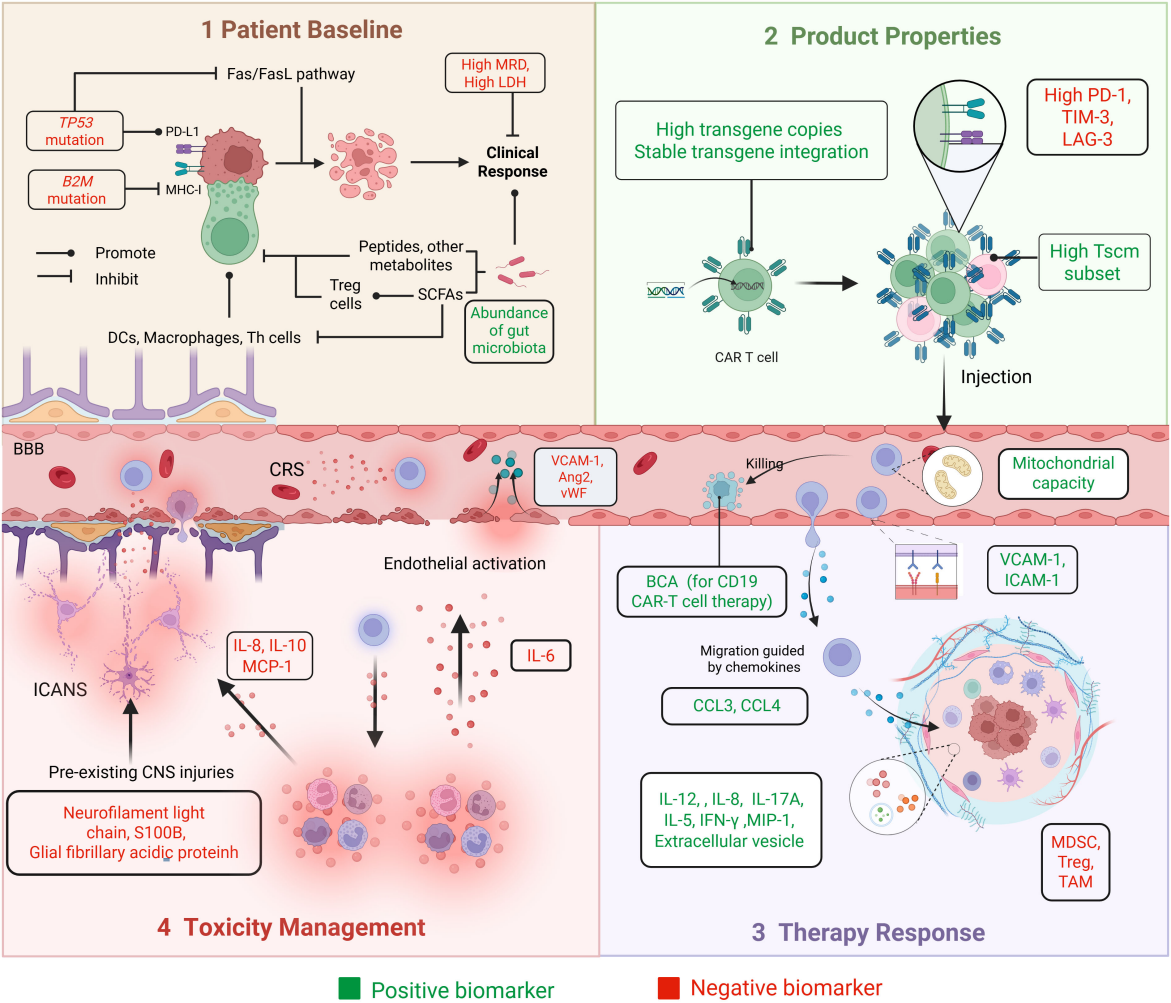
Biomarkers in the main steps of the ACT process. The practical application process of ACT is shown through four panels: Panel 1 (Patient Baseline), Panel 2 (Product Properties), Panel 3 (Therapy Response), and Panel 4 (Toxicity Management). It includes baseline patient assessments, product manufacturing, as well as efficacy and toxicity monitoring. Biomarkers associated with better prognostic outcomes are described as ‘positive biomarkers’, shown in green. Biomarkers associated with poorer prognosis are described as ‘negative biomarkers’, shown in red.

Biomarkers are essential tools in the development and clinical application of ACT, which provide key insights into patient selection, therapeutic efficacy, and toxicity management. They help identify patients who are likely to benefit from ACT, monitor the quality and function of therapeutic cell products, and evaluate long-term outcomes (**Figure 3**)—all of which are crucial for implementing personalized ACT strategies.

### 5.1 Patient baseline

#### 5.1.1 Immunological function and disease burden

Patient-specific factors strongly influence the response to CAR-T therapy. Physiological indicators (e.g., age, heart rate, and blood pressure) and hematologic and biochemical markers, including inflammatory cytokines, leukocyte count, C-reactive protein, hemoglobin, creatinine, ferritin, platelets, and fibrinogen, are essential for evaluating immunological function (171). Disease burden is a key determinant of suboptimal responses after CAR-T therapy (62, 64). Minimal residual disease (MRD), which reflects tumor burden, constitutes a robust predictor of therapeutic response and long-term survival. Patients who achieve MRD–negative complete response (CR) tend to exhibit more favorable outcomes after CAR-T treatment (64). Lactate dehydrogenase (LDH), a key enzyme in the Warburg effect, is associated with tumor burden and metastatic potential. In patients with metastatic melanoma undergoing TIL therapy, elevated LDH levels have been linked to worse prognosis (30, 66).

#### 5.1.2 Genetics

Patients with *TP53* mutations typically exhibit lower remission rates, shorter survival, and reduced responsiveness to combination therapies. *TP53* mutations impair the efficacy of CAR-T therapy through multiple mechanisms, including immune evasion via upregulation of ICP molecules (e.g., PD-L1) and downregulation of MHC-I, inhibition of apoptosis pathways such as Fas/FasL, and suppression of the TME through reduced CD8^+^ T-cell infiltration and downregulation of IFN signaling (172). Zheng et al. investigated concurrent *MYC* abnormalities in the context of *TP53* mutations; patients with dual-positive *MYC* and *TP53* mutations experienced the worst prognoses after CAR-T therapy (173). Shouval et al. reported that *B2M* mutations may result in decreased MHC-I expression, thereby diminishing the ability of CAR-T cells to recognize and eliminate tumor cells (172).

#### 5.1.3 Gut microbiota

The gut microbiota influences immune cell function through its metabolic products, such as short-chain fatty acids (SCFA). These metabolites enhance T-cell effector responses by upregulating the activity of anti-inflammatory Treg cells and CD8^+^ T cells while reducing the activity of pro-inflammatory Mφs, DCs, and Th1/Th17 cells (174, 175). The gut microbiota may also directly modulate T-cell function by secreting peptides and metabolites, which can be influenced via dietary modification or antibiotic administration (174). Patients who achieved CR after CAR-T therapy had greater gut microbiota diversity compared with those who attained only a partial response (PR) (176). Smith et al. observed that patients with antibiotic treatment before CAR-T therapy displayed altered baseline fecal microbiota; they tended to experience decreased survival and increased neurotoxicity (177).

### 5.2 ACT product properties

#### 5.2.1 Stem cell-like properties

Tscm represents a subpopulation of memory T (Tm) cells characterized by self-renewal and multipotency. These cells are capable of differentiating into central Tm (Tcm) cells, effector memory T cells, and Teff cells. Tscm exhibit superior proliferative capacity and more robust immune reconstitution relative to conventional Tm cells (178). In a study, the CD39^-^CD69^-^ Tscm subset was more abundant in CR patients. CD39^-^CD69^-^ cells demonstrated 1,000-fold greater expansion potential than CD39^+^CD69^+^ cells (179).

#### 5.2.2 Immune checkpoints

ICPs can impede ACT by promoting T-cell exhaustion and sustaining an immunosuppressive TME. TILs from certain cancers express high levels of ICP receptors, including PD-1, lymphocyte activation gene 3 (LAG-3), and TIM-3 (143). Among osteosarcoma patients receiving TIL therapy, non-responders exhibited a greater proportion of CD8^+^PD-1^+^ TILs (180). By contrast, among patients with metastatic melanoma, ACT responders displayed higher levels of checkpoint receptors; the expression patterns of PD-1, LAG-3, and TIM-3 may serve as markers for CD8^+^ tumor-reactive T-cell populations within the TIL pool (181).

#### 5.2.3 Transgene integration and expression

The persistence of CAR-T cells, indicated by transgene copy number and CAR-DNA expression, is related to the duration of therapeutic response and long-term survival after CAR-T cell infusion (182). Moreover, the extent of CAR-T cell expansion depends on specific genomic loci where the CAR vector integrates into the patient’s genome (183). Early detection of transgene integration events is crucial to ensure the effective and safe implementation of CAR-T therapy.

### 5.3 Prediction and reflection of therapy response

#### 5.3.1 TME

Various immunosuppressive cell types, such as Treg cells, TAMs, and MDSCs, can infiltrate solid tumors immensely. The activation states of these suppressive cells influence T-cell anti-tumor cytotoxicity. Conversely, cytokines such as IL-12, IFN-γ, macrophage inflammatory protein-1 (MIP-1), IL-8, and IL-17A can counteract immunosuppression (184, 185). For example, IL-12 activates NK cells, promotes the differentiation of CD4+ T cells into IFN-γ-producing Th1 cells, enhances the cytotoxicity of CD8+ T cells, upregulates antigen presentation, and reprograms MDSCs into phenotypes that support T-cell activity (184).

The migration and infiltration of adoptively transferred cells into tumor sites after infusion are critical for anti-tumor efficacy (2). This process is mediated by chemokines such as CCL3 and CCL4, as well as adhesion molecules including vascular cell adhesion molecule-1 (VCAM-1) and ICAM-1, and other guidance cues (48). A deficiency in these molecules may predict suboptimal CAR-T cell expansion and poor treatment outcomes.

#### 5.3.2 T-cell activity and function

CD19 is mainly expressed on B cells; CD19 CAR-T therapy leads to B-cell clearance, resulting in B-cell aplasia (BCA). BCA serves as a biomarker of CAR-T cell activity and is associated with sustained remission. The duration and recovery of BCA are correlated with CAR-T cell persistence, disease burden, and subsequent treatment interventions (186). In a multi-institutional retrospective study, early B-cell recovery (occurring within 6 months) was associated with a higher risk of CD19^+^ leukemia relapse (187).

After infusion, CAR-T cells expand, migrate, recognize target cells, and execute cytotoxic functions. These processes require sufficient energy production by mitochondria, and successful mitochondrial remodeling is essential. Failure to shift metabolic activities may result in prolonged glycolysis, impaired energy production, and the development of ineffective, short-lived CAR-T cells (188). Extracellular vesicles (EVs), membrane-derived particles that facilitate intercellular communication, also help to regulate T-cell function. A higher concentration of CD69^+^ T cell–derived vesicles has been associated with enhanced T-cell activation and limitation of excessive T-cell stimulation (189).

### 5.4 Toxicity management

CRS and ICANS are the most common severe toxicities after CAR-T therapy. Endothelial activation plays a central role in their pathogenesis (190). Teachey et al. developed predictive models using cytokine levels, tumor burden, and other variables to estimate the likelihood of grade 4–5 CRS (191). Hong et al. constructed a decision tree model incorporating soluble VCAM-1, the angiopoietin (Ang)-2:Ang-1 ratio, and soluble ICAM-1 to predict CRS occurrence and severity (190). IL-6 is regarded as a cytokine involved in endothelial permeability and CRS development. IL-6 receptor inhibitors, such as tocilizumab, may be administered before the IL-6 level peaks, guided by predictive modeling (192).

ICANS occurring after CAR-T infusion may be linked to blood–brain barrier disruption and increased permeability (193). Platelets release Ang-1, which stabilizes endothelial cells; thus, thrombocytopenia contributes to capillary leakage and is associated with severe CRS and ICANS (194). A high Ang-2:Ang-1 ratio and elevated von Willebrand factor (vWF) levels have been observed in patients with grade ≥4 ICANS (194). ICANS-related dysregulation can also be reflected by cerebrospinal fluid (CSF) cytokine levels, including IL-8, IL-10, and monocyte chemoattractant protein-1 (MCP-1) (35, 195). Biomarkers of CNS injury measurable before CAR-T infusion, such as neurofilament light chain, S100 calcium-binding protein B (S100B), and glial fibrillary acidic protein, are detectable in blood and directly reflect their CSF concentrations. These biomarkers may serve as predictors of high-grade ICANS (193, 196, 197).

### 5.5 Clinical decision-making in combination therapy

#### 5.5.1 ACT + ICIs

ICP signaling promotes T-cell exhaustion and contributes to ACT failure (3). Exhaustion marker expression patterns may guide the use of ICI therapy, which can reverse exhaustion-related dysfunction (198). In a phase 1/2a study (198), 12 participants received pembrolizumab after CAR-T therapy failure. Among the three responders, all had tumors with PD-L1 expression exceeding 5%. By contrast, non-responders exhibited elevated levels of exhaustion biomarkers, such as thymocyte selection-associated high-mobility group box (TOX), CD57, and T-cell immunoreceptor with immunoglobulin and ITIM domain (TIGIT); responders, on the other hand, demonstrated higher levels of activation and proliferation markers, including CD26, CD127, and CD69.

MDSC level has been identified as an independent negative prognostic factor for survival in melanoma patients (199). A proposed approach involves administering anti-MDSC therapy with NKG2D.ζ-engineered NK cells prior to ICI treatment (144). Therefore, baseline MDSC levels constitute candidate biomarkers for selecting patients who may benefit from combined NK cell and ICI therapy.

#### 5.5.2 ACT + RT/chemotherapy

Biomarkers can assist in determining the appropriate dose of RT and whether RT or chemotherapy should be supplemented to improve clinical outcomes. Amit et al. treated pancreatic cancer patients with proton RT followed by mesothelin CAR-T therapy. Their findings indicated that proton radiation enhanced the efficacy of mesothelin CAR-T therapy (200). Accordingly, higher radiation doses or extended RT cycles may be warranted in mesothelin CAR-T therapy for patients exhibiting low mesothelin expression.

A predominant pattern of CAR-T treatment failure is disease progression at pre-existing sites, particularly in patients with a high tumor burden. RT can effectively control localized lesions and reduce local recurrence risk after CAR-T therapy (201). Saifi et al. reported that patients with localized disease, elevated disease burden, high LDH levels, and extra nodal invasion had worse prognoses and often underwent RT before CAR-T cell infusion (201). Shi et al. used magnetic resonance imaging and alpha-fetoprotein (AFP) levels to evaluate residual lesions after RT in hepatocellular carcinoma (HCC). To prevent relapse and metastasis, patients subsequently received GPC3 CAR-T therapy, resulting in substantial reductions in AFP fetoprotein levels (151).

Zhao et al. (149) performed phenotypic analysis of peripheral blood mononuclear cells before and after combination therapy with DC/CIK cells and chemotherapy in patients with non-small cell lung cancer (NSCLC). They found that the proportion of CD8^+^CD28^-^ T cells was negatively correlated with PFS and OS. These findings suggest that patients with a high percentage of CD8^+^CD28^-^ T cells require more intensive treatment regimens, such as increased chemotherapy dose or frequency, or combination with other immunotherapies.

## 6 Discussion

ACT has emerged as a transformative cancer immunotherapy, using engineered immune cells for targeted tumor killing. FDA-approved approaches show durable responses in hematologic and solid tumors, and other novel ACTs are also under research. However, efficacy of ACT is limited by challenges like immunosuppressive TME, poor cell trafficking and infiltration, cell exhaustion, tumor heterogeneity, and antigen escape, driving the need for combination strategies and biomarker-guided optimization.

Although this article provides a brief overview of the challenges associated with ACT, further research is required to elucidate the underlying mechanisms. Within the TME, synergistic interactions among immunosuppressive cell populations and the impacts of tumor-derived metabolites on immune cells are not fully understood. The precise effects of tumor vascular abnormalities on immune cell trafficking, as well as the dynamic regulation of chemokine networks during immune cell transport and infiltration, also require clarification. Additional key areas to investigate include early indicators of T-cell exhaustion, evolving patterns of antigenic heterogeneity, specific epigenetic mechanisms that govern antigen escape in tumor cells, the role of cancer stem cells, and the influence of microenvironmental heterogeneity on tumor behavior.

Combination therapy strategies are essential for overcoming the limitations of ACT. When ACT is combined with ICIs, chemotherapy, or RT, inhibitory factors such as ICP signaling pathways and MDSCs may be attenuated. Tumor vaccines enhance ACT efficacy by broadening the antigenic spectrum for adoptive immune cells. Furthermore, cytokine modulation with ACT preserves Tscm phenotypes, prolonging therapeutic activity. Concurrently, biomarker-guided monitoring supports the early identification and management of toxicities such as CRS. Combination regimens can strengthen ACT in multiple ways. For example, RT upregulates chemokines and adhesion molecules within the tumor vasculature, enhancing ACT cell homing. Overall, these strategies establish a comprehensive framework to enhance ACT efficacy and accelerate its clinical translation by simultaneously targeting multiple barriers. Successful combination therapy requires the incorporation of expertise across diverse fields. This multidisciplinary collaboration must be supported by efficient communication and resource integration to ensure optimal treatment implementation.

Biomarkers are critical for optimizing ACT at various stages. Baseline factors, such as TMB and genetic profiles, predict clinical benefit. Product-related biomarkers ensure optimal cell preparation selection, while multiple molecules, like cytokines and chemokines, indicate therapy efficacy. Furthermore, Biomarker-guided monitoring aids early CRS and ICANS management. Yet, validating biomarker utility in clinical trials is complex, requiring strict regulation. However, the design of rational clinical trials to validate biomarker utility remains a complex process. Regulatory agencies impose stringent requirements for demonstrating the clinical validity, safety, and quality control of biomarker-based strategies. Future research should prioritize cell–cell interactions and cytokine/chemokine regulation within the TME. There is also a need to develop technologies that allow real-time monitoring of antigenic heterogeneity. Integration of these approaches with multi-omics platforms may support the development of personalized therapeutic strategies.

The application of ACT is becoming increasingly broader, and has extended to non-malignant conditions like lupus erythematosus (202). However, its widespread implementation remains constrained by the time- and cost-intensive manufacturing. Off-the-shelf ACT products offer a promising alternative for this, and ongoing research of allogeneic CAR-T products is advancing this field (203). A recent clinical study utilizing an AI model to assist TCR identification showed encouraging outcomes in treating solid tumors. With the development of off-the-shelf ACT products and the deepening of AI-clinical integration, the application of individualized ACT based on combination therapies and biomarkers holds a promising future.

## Glossary

ACT: adoptive cell therapy
AFP: alpha-fetoprotein
ALL: acute lymphoblastic leukemia
AML: acute myeloid leukemia
Ang: angiopoietin
APC: antigen-presenting cell
B-ALL: B-cell acute lymphoblastic leukemia
BBB: blood-brain barrier
BCA: B cell aplasia
BCMA: B-cell maturation antigen
CAR: chimeric antigen receptor
CAR-M: CAR-macrophage
CCL: chemokine (C-C motif) ligand
CD: cluster of differentiation
CD40L: CD40 ligand
cGAS-STING: cyclic guanosine monophosphate/adenosine monophosphate synthase–
CI: confidence interval
CIK: cytokine-induced killer
CLDN6: Claudin 6
CMV: cytomegalovirus
CNS: central nervous system
CR: complete response
CRC: colorectal cancer
CRR: complete response rate
CRS: cytokine release syndrome
CSF: cerebrospinal fluid
CTL: cytotoxic T lymphocytes
CXCL: chemokine (C-X-C motif) ligand
DAMPs: damage-associated molecular patterns
DC: dendritic cell
DFS: disease-free survival
DLBL: diffuse large B-cell lymphoma
DOR: Date of response
EFS: event-free survival
EGFR: epidermal growth factor receptor
EGFRvIII: epidermal growth factor receptor variant III
Eps8: epidermal growth factor receptor pathway substrate 8
EVs: extracellular vesicles
FDA: US Food and Drug Administration
GD2: glycoprotein D2
GFAP: glial fibrillary acidic protein
GM-CSF: granulocyte-macrophage colony-stimulating factor
GPC3: glypican-3
HCC: hepatocellular carcinoma
HER2: human epidermal growth factor receptor 2
HMGB1: high-mobility group box 1
HNSCC: head and neck squamous cell carcinoma
ICAM-1: intercellular adhesion molecule-1
ICANS: immune effector cell-associated neurotoxicity syndrome
ICD: immunogenic cell death
ICI: immune checkpoint inhibitor
ICP: immune checkpoint
ICR: inverted cytokine receptor
IFN: interferon
IL: interleukin
IL-21R: interleukin-21 receptor
IL-4R: interleukin-4 receptor
IL-7R: interleukin-7 receptor
iMACs: induced pluripotent stem cells
KIR: killer immunoglobulin-like receptors
LAG-3: lymphocyte activation gene 3
LBCL: large B-cell lymphoma
LDH: lactate dehydrogenase
LD-TBI: low-dose total body irradiation
MCP-1: monocyte chemoattractant protein 1
MHC: major histocompatibility complex
MIP-1: macrophage inflammatory protein-1
MM: multiple myeloma
MMPs: matrix metalloproteinases
mo: month
mOS: median overall survival
mPFS: median progression-free survival
MPM: malignant pleural mesothelioma
MRD: minimal residual disease
mRNA: messenger RNA
Mφ: macrophage
N/A: not applicable
NCT: national clinical trial
NfL: neurofilament light chain
NHL: non-Hodgkin lymphoma
NK: natural killer
NKG2D.ζD-NK: NKG2D.ζD-natural killer
NR: not reached
OR: overall response
ORR: objective response rate
OS: overall survival
OX40L: OX40 ligand
PD: progressive disease
PD-1: programmed cell death protein 1
PD-L1: programmed death-ligand 1
PFS: progression-free survival
pp65: phosphoprotein 65
PR: partial response
PSCA: prostate stem cell antigen
PTCL: peripheral T-cell lymphoma
pt: patient
r/r: relapsed/refractory
Ref: reference
RNA-LPX: RNA-lipoplexes
ROS: reactive oxygen species
RT: radiotherapy
S100B: S100 calcium-binding protein B
SCFA: short-chain fatty acids
scFv: single-chain variable fragment
SD: stable disease
TAA: tumor-associated antigen
Tcm: central memory T
TCR: T-cell receptor
Teff: effector T
TGF-β: transforming growth factor-beta
Th1: T helper type 1
Th17: T helper type 17
TIGIT: T-cell immunoreceptor with immunoglobulin and ITIM domain
TIL: tumor-infiltrating lymphocyte
TIM-3: T-cell immunoglobulin and mucin domain 3
TIR: Toll/IL-1 receptor
TMB: tumor mutational burden
TME: tumor environment
TNF: tumor necrosis factor
TOX: thymocyte selection-associated high-mobility group box
TRAIL-R2: tumor necrosis factor-related apoptosis-inducing ligand receptor 2
TRBC1: T-cell receptor beta chain 1
Treg: regulatory T
TSA: tumor-specific antigen
Tscm: stem cell-like memory T-cells
VCAM-1: vascular cell adhesion molecule-1
wks: weeks
WT1: Wilms’
Yr: year
